# A New Method for the Dynamics Analysis of Super-Elastic-Plastic Foams under Inhomogeneous Loading and Unloading Conditions

**DOI:** 10.3390/polym16172489

**Published:** 2024-08-31

**Authors:** Jiaxuan Chen, Fude Lu, Mingqi Wang, Shuangxi Xiang

**Affiliations:** School of Packaging and Material Engineering, Hunan University of Technology, Zhuzhou 412007, China

**Keywords:** residual deflection, polyethylene foam, finite element modeling, indentation test, inhomogeneous compression

## Abstract

In this research, a new computational method was proposed for describing the mechanical behavior of super-elastic-plastic foams under inhomogeneous compressive impacts. The method regarded the foam material as composed of two typical mechanical properties superimposed multiple times: one was the hyper-elastic layer, and the other was the elastoplastic layer. The hyper-elastic layer and the elastoplastic layer were interwoven and overlapped, divided into double-layer, four-layer, and six-layer configurations to characterize the foam material. After the equivalent layering of the foam, by comparing the results of the four-layer and six-layer divisions, it was found that when the layering reached four layers, the foam performance curve had already converged. The study utilized the HYPERFOAM model and Mullins effect in the ABAQUS software to establish the constitutive relationship of the hyper-elastic layer. It adopted the Crushable foam model to develop the constitutive relationship of the elastoplastic layer. Under uniaxial compression conditions, quasi-static and intermediate strain rate compression tests were performed on polyethylene (PE) foam materials with three different densities. Based on the experimental results, the parameter values of the hyper-elastic-plastic foam model in the ABAQUS code were determined. By comparing the computational results and the experimental results, the established finite element (FE) model was validated using the mechanical behavior of indentation and compression tests. The results showed that this method could effectively describe the complex mechanical behavior and residual deformation of hyper-elastic-plastic foam packaging materials under non-uniform compression, and the experimental and simulation results agreed well, proving the reliability of this method.

## 1. Introduction

The most commonly utilized cushioning material is polyethylene (PE) foam. This is because as a super-elastic-plastic foam material, polyethylene (PE) foam possesses a composite mechanical response that amalgamates the attributes of super-elasticity and plasticity. This allows it to demonstrate super-elastic behavior during the initial stages of deformation, followed by plastic behavior upon experiencing greater degrees of deformation—a property that makes it a widely utilized cushioning material. This material also exhibits lightweight and high-strength characteristics, in addition to remarkable energy absorption and cushioning capabilities. It is extensively used as a cushioning material in transport packaging applications [1]. The cushioning curve, especially the maximum acceleration-static stress curve, is utilized to design cushion sizes by engineers, but it requires a highly tedious construction process [2]. Ruiz-Herrero [3,4] introduced adiabatic and isothermal models incorporating the air effect to predict maximum acceleration-static stress curves for low-density PE foam. According to the method of the dynamic factor proposed by Sek [5], Navarro-Javierre [6] and Li [7] deduced the cushioning curves of PE foam. In the literature [6], the Burgess equation fit method was also utilized to evaluate the cushioning curves of PE foam based on the relationship between dynamic stress and energy density. Lu [8,9] proposed a phenomenological constitutive relationship for PE foam based on quasi-static and dynamic stress–strain data, which can predict the acceleration responses of an object dropped onto a PE foam cushion by solving vibration equations. All of the above results significantly reduce the time to characterize the mechanical properties of PE foam. Based on the Odgen hyper-elastic model, Jebur [10] used the HYPERFOAM model to investigate the uniaxial compression, but complex deformation was not involved. On the other hand, the HYPERFOAM model is also applicable to other resilient foam, such as polyurethane foam [11] and polypropylene foam [12].

According to the above studies, we can find that it is not suitable for corner, edge, and end foam structures in real-world packaging. Ge [13] reported the cushion curves of the corner PE pad that were compared with the flat foam, showing the average static stress for the corner foam during the compression hysteresis test was more than 23%.

However, few examples in the literature have been found regarding complex deformation analysis for PE foam, Mills [14] applied a Crushable foam model to investigate a corner and end cap foam structure made of PE foam, but it provided no information on the mechanical responses about the unloading phase, which was due to the fact that crushable foam was found not to match the foam unloading response [15,16,17]. The residual deformation for PE foam is about 0.2, meaning the 80% deformation can be recovered with unloading. The hyper-elastic model and the Mullins effect model are successfully applicable to investigate the resilient polypropylene foam.

In this work, a novel computational method was proposed to characterize the mechanical behavior of plastic foams under non-uniform compressive impacts. This approach viewed the foam as consisting of two distinct foam mechanical property regions: an elastic-plastic layer and a hyper-elastic layer. During the loading phase, both the elastic-plastic layer and the super-elastic layer exhibited the same mechanical response. However, during the unloading phase, the elastoplastic layer demonstrated linear elastic behavior, while the super-elastic layer exhibited nonlinear action. The mechanical behavior of the foam under heterogeneous compression impact was described by superimposing the unique mechanical responses of these two layers multiple times.

For complex foam structures, a finite element model is essential. The foam constitutive models in the ABAQUS code provide powerful tools for analysis of large deformation of resilient PE under the uniaxial compressive loading situation [9]. The aim of this study was to model a corner cushion made of PE foam by using ABAQUS software, which is applicable to loading and unloading, and providing an effective way to realize the scientific design.

## 2. Materials and Methods

PE foam, which had three different densities of 29 kg/m^3^, 36 kg/m^3^, and 58 kg/m^3^, was investigated for its mechanical properties. The latter two foams were manufactured by Dow Chemistry Company and were referred to as EPE36 and EPE58. The 29 kg/m^3^ density foam was manufactured by expanded processing and was referred to as EPE29. For each sample size, three repeat tests were performed to avoid data dispersion.

As we know, PE foam exhibits a rate dependency to a certain extent. Thus, the intermediate strain rate tests were needed in addition to quasi-static tests. Quasi-static compression tests and drop impact tests were undertaken by using a universal machine and a drop tower machine, respectively.

**Figure 1:**
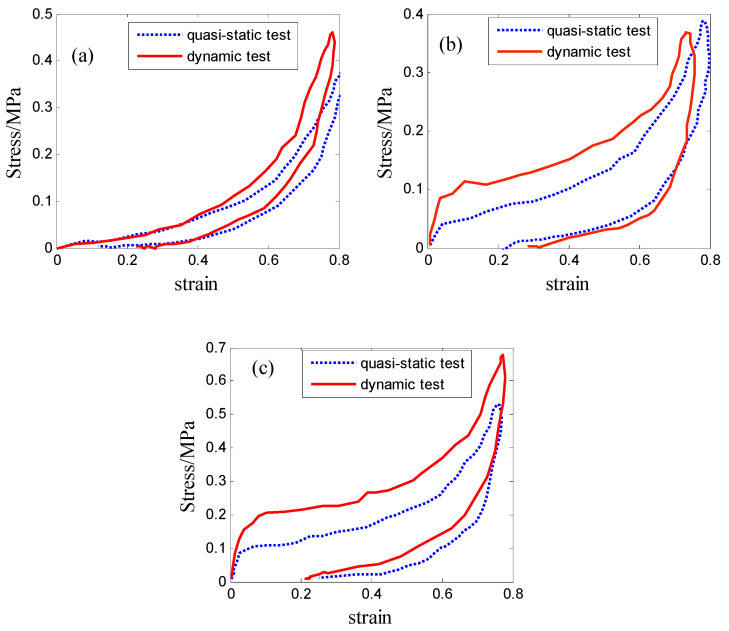
Stress–strain curve of PE foam: (**a**) EPE29, (**b**) EPE36, and (**c**) EPE58.

The foam dimensions of 10 cm × 10 cm × 5 cm were chosen for the 29 kg/m^3^ foam. The compressive velocity of 30 mm/min was applied for the compressor, corresponding to the compressive strain rate of 0.01 s^−1^. The same foam dimension was subjected to a free drop impact of the mass of 7.5 kg from a height of 60 cm. The engineering stress–strain curves could be transformed by twice integrating the acceleration–time signals. Figure 1a compares the quasi-static stress–strain curve with the dynamic stress–strain curve for the loading and unloading phases. It illustrates that the value of dynamic stress was higher than the quasi-static counterpart when the strain was bigger than 0.4. As can also be seen, the stress was almost the same when the strain was less than 0.4.

The foam dimensions of 10 cm × 10 cm × 4 cm were chosen for EPE36. The compressive velocity of 25 mm/min was applied for the compressor, corresponding to a compressive strain rate of approximately 0.01 s^−1^. A weight of 7.5 kg and drop heights of 70 cm were used for this type of PE foam. Figure 1b shows a comparison of static stress–strain data with dynamic stress–strain data. It can be observed that the strain rate played an important role in compressive behaviors.

The foam dimensions of 10 cm × 10 cm × 3 cm were chosen for EPE58. The compressive velocity of 20 mm/min was applied for the compressor, corresponding to a compressive strain rate of approximately 0.01 s^−1^. A weight of 7.5 kg and drop heights of 90 cm were used for this type of PE foam. Figure 1c shows the relationship between static and dynamic stress–strain data. It demonstrates the same phenomenon, in which the strain rate was essential to the compressive behaviors.

The discussion above implies that EPE36 and EPE58 PE foams exhibited sensitivity to the strain rate and had three distinctive phases, i.e., linearity, stress plateau phase, and densification phases. It was seen that EPE29 demonstrated different compressive behaviors compared with the other PE foam. EPE29 foam exhibited tangent nonlinear mechanical properties without the stress plateau region.

## 3. Basic Theory of Super-Elastic-Plastic Foam Modeling

### 3.1. The Layered Structure of Super-Elastic-Plastic Foams

**Figure 2:**
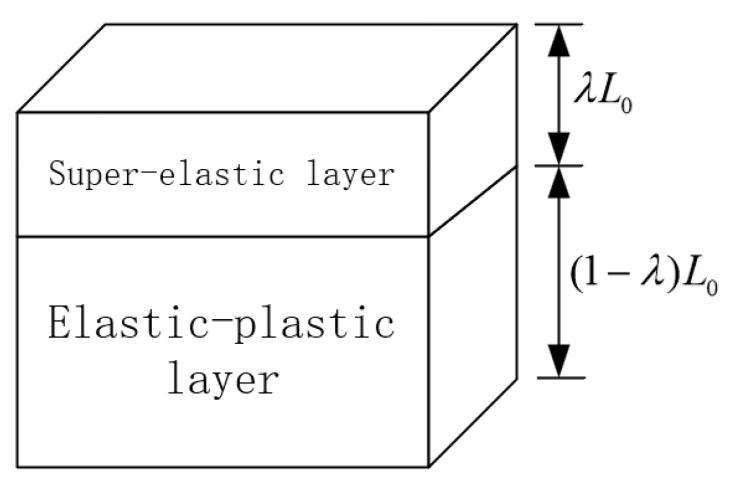
Equivalent diagram of foam mechanical properties.

The treatment of the foam as a tandem of the mechanical properties of two typical foams is schematically shown in Figure 2. The two foam layers possessed identical cross-sectional areas but differing thicknesses, assuming the thicknesses of the respective portions were λL_0_ and (1−λ)L_0_, where L_0_ denotes the original total thickness. The ratio of the thickness of the super-elastic layer to the elastic-plastic layer was maintained as a constant λ/(1−λ). On this basis, the mechanical properties of foam in inhomogeneous compression were investigated, and the foam was regarded as a multilayer stack of two typical properties.

### 3.2. Super-Elastic-Plastic Foam Constitutive Model

The HYPERFOAM model and the Mullins model developed in ABAQUS simulated the non-linear behavior of the loading and unloading phases of a super-elastic foam hyper-elastic layer. The Ogden strain energy function U used to guide the HYPERFOAM model can be expressed [18] as

(1) $$U(\lambda_{1},\lambda_{2},\lambda_{3})=\sum_{i=1}^{N}\frac{2\mu_{i}}{\alpha_{i}^{2}}\{\lambda_{1}^{\alpha_{i}}+\lambda_{2}^{\alpha_{i}}+\lambda_{3}^{\alpha_{i}}-3+\frac{1}{\beta_{i}}((\lambda_{1}\lambda_{2}\lambda_{3}{)}^{-\alpha_{i}\beta_{i}}-1)\}$$

where $\mu_{i}$, $\alpha_{i}$, and $\beta_{i}$ are material parameters. $\lambda_{i}$ (i = 1, 2, 3) are principle stretches.

Because it was assumed to be positive in compression, one-dimensional stress should be written as follows:

(2) $$\sigma =\sum_{i=1}^{N}\frac{2\mu_{i}}{\alpha_{i}(1-\varepsilon )}[1-{\left(1-\varepsilon \right)}^{\alpha_{i}}]$$

Therefore, the stress for the uniaxial state for the unloading process is written as

(3) $$\bar{\sigma }=\{1-\frac{1}{r}\mathrm{erf}(\frac{U_{m}-U_{0}}{m_{1}+\beta U_{m}})\}\sigma$$

where r, *m*_1_, and β are material constants. Under uniaxial stress state, strain energy ($U_{0}$) is

(4) $$U_{0}=\sum_{i=1}^{N}\frac{2\mu_{i}}{\alpha_{i}}[{\left(1-\varepsilon \right)}^{\alpha_{i}}-\ln(1-\varepsilon )]$$

and maximum strain energy ($U_{m}$) is

(5) $$U_{m}=\sum_{i=1}^{N}\frac{2\mu_{i}}{\alpha_{i}}[{\left(1-\varepsilon_{m}\right)}^{\alpha_{i}}-\ln(1-\varepsilon_{m})]$$

For the elastic-plastic layer, the mechanical response during the loading phase was the same as that of the hyper-elastic layer. However, during the unloading stage, the behavior was characterized by linear elastic unloading, which could be represented by the Crushable foam model developed in ABAQUS. Therefore, the constitutive relationship for the elastic-plastic layer is

(6) $$\begin{array}{l} \sigma =\frac{2\mu_{1}}{\alpha_{1}(1-\varepsilon )}[1-(1-\varepsilon {)}^{\alpha_{1}}]+\frac{2\mu_{2}}{\alpha_{2}(1-\varepsilon )}[1-(1-\varepsilon {)}^{\alpha_{2}}]\;\;\;\;\dot{\varepsilon }\geq 0\;\\ \sigma =E(\varepsilon -\varepsilon_{m})+\sigma_{m}\;\;\;\;\;\;\;\dot{\varepsilon }<0 \end{array}$$

when the maximum compression strain ε_m_ was reached.

The material parameters for the strain energy of Equation (2) were determined based on the loading stress–strain data shown in Figure 1, while r, m, β, **λ**, and the modulus of elasticity E were determined by the unloading stress–strain data. The nonlinear iteration function LSQNONLIN was used by MATLAB, and the identified parameter results are summarized in Table 1.

Under uniaxial loading, the constitutive relationship for the super-elastic-plastic foam was as follows:

Super-elastic layer ontological relationships:

(7) $$\sigma =\frac{2\mu_{1}}{\alpha_{1}(1-\varepsilon )}[1-{\left(1-\varepsilon \right)}^{\alpha_{1}}]+\frac{2\mu_{2}}{\alpha_{2}(1-\varepsilon )}[1-{\left(1-\varepsilon \right)}^{\alpha_{2}}]\;\;\;\dot{\varepsilon }\geq 0\;$$

(8) $$\sigma =\{\frac{2\mu_{1}}{\alpha_{1}(1-\varepsilon )}[1-{\left(1-\varepsilon \right)}^{\alpha_{1}}]+\frac{2\mu_{2}}{\alpha_{2}(1-\varepsilon )}[1-{\left(1-\varepsilon \right)}^{\alpha_{2}}]\}\{1-\frac{1}{r}\mathrm{erf}(\frac{U_{m}-U_{0}}{m_{1}+\beta U_{m}})\}\;\;\;\dot{\varepsilon }<0$$

Elastic-plastic layer principal relationships:

(9) $$\begin{array}{l} \sigma =\frac{2\mu_{1}}{\alpha_{1}(1-\varepsilon )}[1-(1-\varepsilon {)}^{\alpha_{1}}]+\frac{2\mu_{2}}{\alpha_{2}(1-\varepsilon )}[1-(1-\varepsilon {)}^{\alpha_{2}}]\;\;\;\;\dot{\varepsilon }\geq 0\;\\ \sigma =E(\varepsilon -\varepsilon_{m})+\sigma_{m}\;\;\;\;\dot{\varepsilon }<0 \end{array}$$

**Figure 3:**
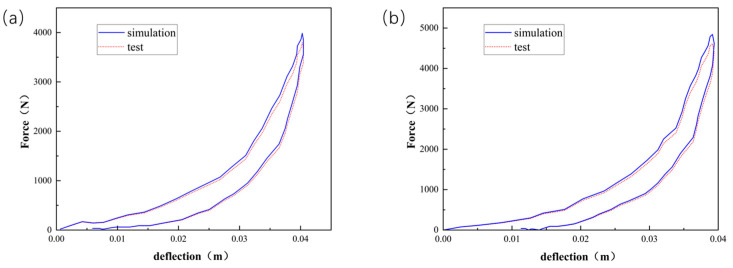
Simulations and compressive tests of EPE29: (**a**) static loading and (**b**) dynamic loading.

**Figure 4:**
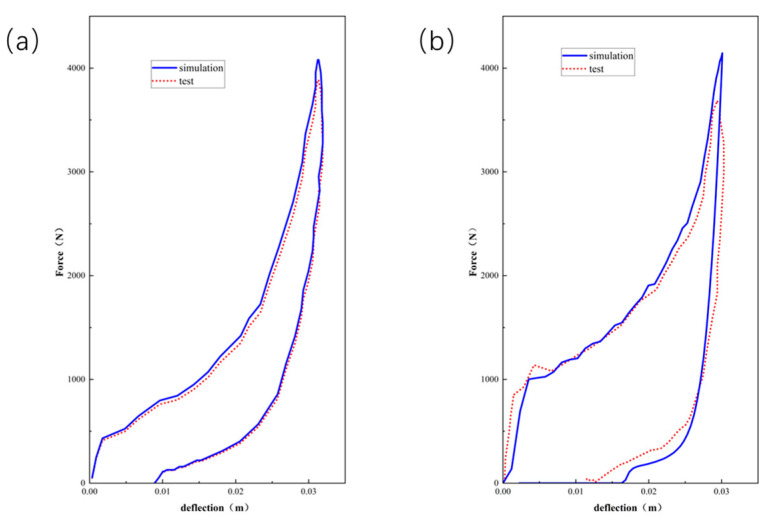
Simulations and compressive tests of EPE36: (**a**) static loading and (**b**) dynamic loading.

**Figure 5:**
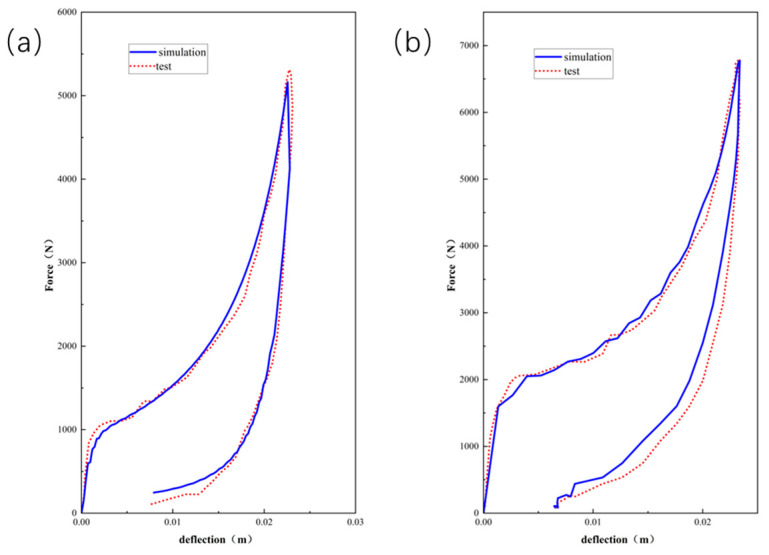
Simulations and compressive tests of EPE58: (**a**) static loading and (**b**) dynamic loading.

Employing Equations (7) and (9), Figure 3, Figure 4 and Figure 5 compare the simulated force–deflection curves with the experimental data for the EPE29, EPE36, and EPE58 PE foam samples under uniaxial loading and unloading conditions. This comparison served to verify the correctness of the parameter values listed in Table 1.

### 3.3. Validation of PE Foam Model

For the three-dimensional situations, the stress is

(10) $$\sigma_{ij}=\frac{\lambda_{i}}{J}\frac{\partial U(\lambda_{1},\lambda_{2},\lambda_{3})}{\partial \lambda_{j}}$$

**Figure 6:**
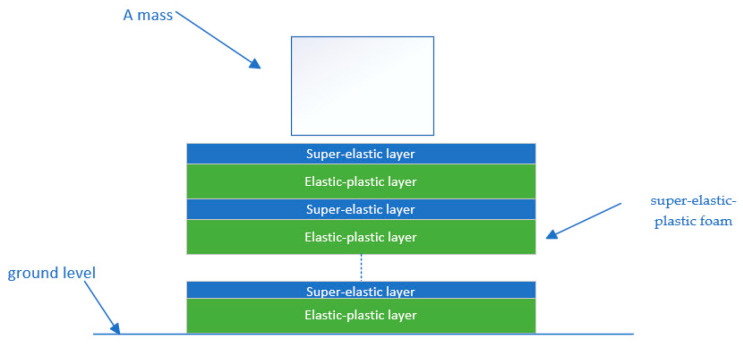
Finite element model of a trapezoid structure.

The HYPERFORM and Mullins models in ABAQUS and the Crushable foam model were used to investigate the response of polyethylene foam in the case of indentation. The indentation tests were as follows: (1) The polyethylene foam specimen size of EPE29 was 10 cm × 10 cm × 5 cm, that of EPE36 was 10 cm × 10 cm × 4 cm, and that of EPE58 was 10 cm × 10 cm × 3 cm, respectively, and a rectangular compressor with a size of 10 cm × 4.5 cm × 3 cm was used for the test. (2) The mesh type of CPE4R and a 1 mm mesh size were selected to grind the foam. (3) A general contact option was made to avoid material penetrating with each other. (4) The rectangular indenter was modeled as a rigid body with a 1 mm mesh size. The constant compressive velocity of 0.5 mm/s was applied to the reference point of the rigid body. (5) The vertical freedom of the rigid body was free, and the other freedom was fixed, while all freedoms were fixed for the beam part to simulate the floor. (6) The friction coefficient between the foam and beam parts was set to 0.3. (7) The foam material was modeled as a layered structure in ABAQUS, divided into two distinct mechanical property profiles overlapping multiple times, as shown in Figure 6.

**Figure 7:**
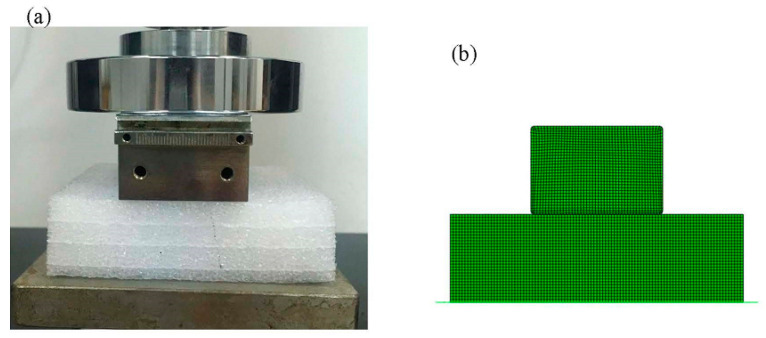
Compressive tests (**a**) and simulations (**b**) of EPE foam.

**Figure 8:**
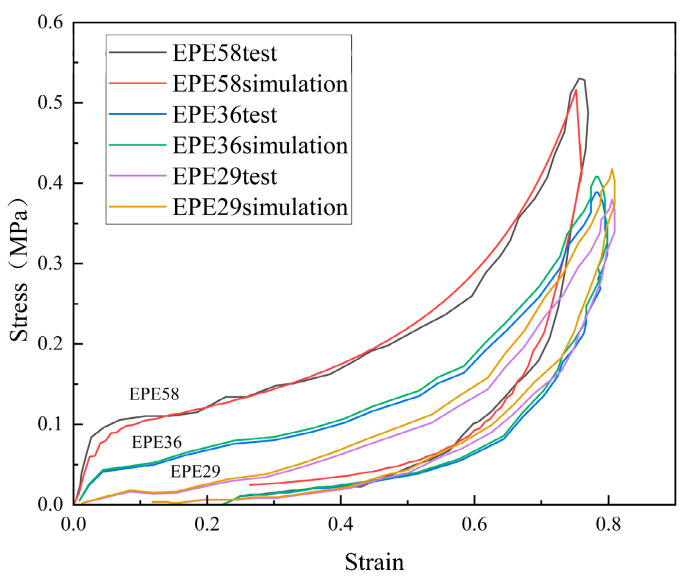
Comparison of stress–strain curves between simulations and compressive tests of PE foam.

**Figure 9:**
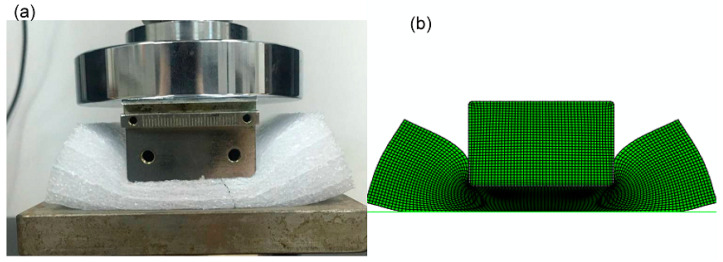
Comparison of deformations between compressive tests (**a**) and simulations (**b**) of EPE58 at strain of 0.7.

Figure 7 represents the initial state of EPE foam during the simulation and testing process.The simulated stress–strain result of EPE foam is presented in Figure 8, and the deformed shapes of 70% strain are shown in Figure 9. The above two figures show that the finite element simulations of the super-elastic-plastic foams model were consistent with the experimental results.

**Figure 10:**
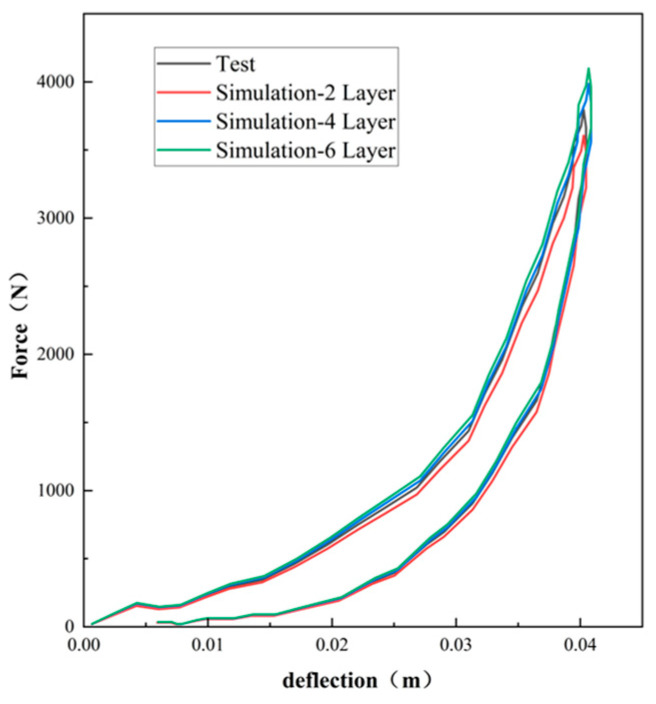
Comparison results between indentation and compressive simulation of EPE29 with force–deflection curves.

**Figure 11:**
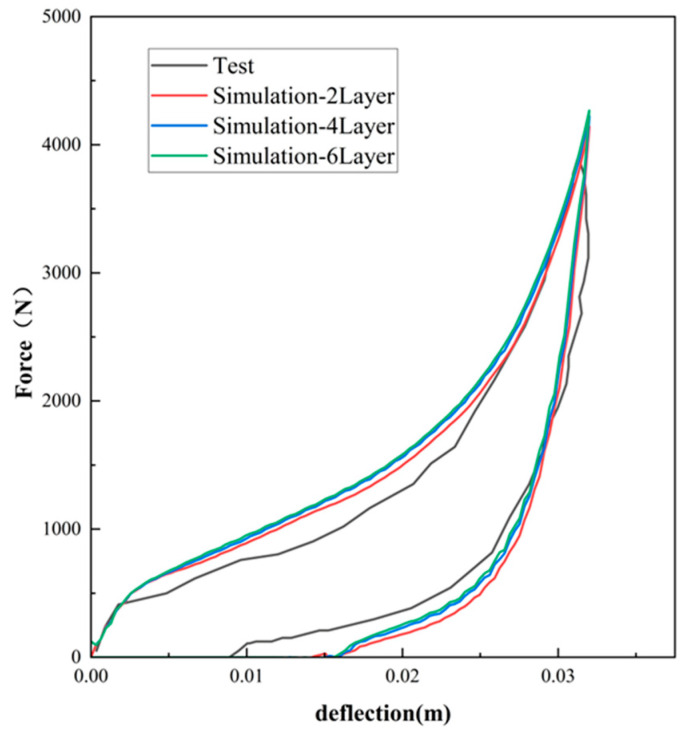
Comparison results between indentation and compressive simulation of EPE36 with force–deflection curves.

**Figure 12:**
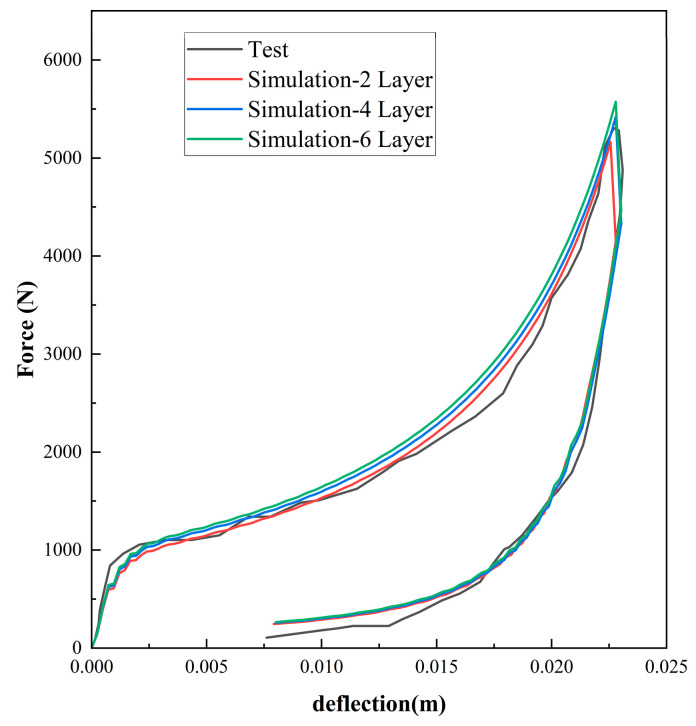
Comparison results between indentation and compressive simulation of EPE58 with force–deflection curves.

As can be seen from Figure 10, Figure 11 and Figure 12, the experimental results are in good agreement with the finite element simulation results, which proved the validity of the layered modeling method. This indicated that describing the foam material in terms of a layered structure with two typical properties intertwined and superimposed could accurately capture its mechanical response under inhomogeneous compression conditions.

Compared with other modeling approaches, the proposed hierarchical model showed excellent results in predicting the residual deformation of plastic foam materials under non-uniform compressive impacts. The model could predict more accurately the complex plastic deformation behavior of foams subjected to local compressive loading. Specifically, the hierarchical modeling approach could accurately simulate the situation where the foam underwent significant plastic deformation in the loaded region and remained relatively intact in the unloaded region. The local deformation characteristics were highly consistent with the deformation mechanism of the actual foam material under non-uniform compression, which demonstrated the ability of the model to capture the complex mechanical behavior of foams.

In terms of modeling with Abaqus software, the classical HYPERFORM and Crushable foam models in Abaqus software were adopted to describe the mechanical response of the foam, which had the advantage of simpler implementation than some complex foam constitutive models. At the same time, fewer material parameters were required, which were easy to obtain from experiments and input into the finite element model.

In Abaqus finite element simulations, modeling the foam as a multilayer structure with two performance characteristics superimposed on each other did not mean that more layers were better. Comparing the simulation results of four and six layers, it was found that they basically overlapped, which indicated that the mechanical properties of the foam had already been fully equated at this point, and increasing the number of layers would only increase the unnecessary computational workload without improving the prediction accuracy of the model.

## 4. Conclusions

Quasi-static and intermediate strain rate tests were carried out on polyethylene foams at three different densities. The models, i.e., HYPERFOAM and Mullins constitutive relations, with built-in Abaqus and parameter values for the Crushable foam model were then determined. The finite element foam model was validated by predicting the 2D indentation response. Finally, the three-dimensional structure of polyethylene foam was tested for impact performance. The conclusions were as follows:

(1) The stress considerably increased with the increasing strain rate for uniaxial compressive tests with a strain rate range from 0.01 to 100 s^−1^ for EPE36 and EPE58 PE foams. However, EPE29 exhibited low rate dependency compared with EPE36 PE under almost the same density. The lack of a plateau region was due to less contribution of the cell structure that was sensitive to the compressive strain rate.

(2) The mechanical response of foam structures could be predicted by using the HYPERFOAM and Mullins constitutive relations to represent the super-elastic layers and by using the Crushable foam model to represent the elastic-plastic layers overlapping multiple times.

It can be seen that modeling the foam material as a layered structure consisting of two classical property characteristics intertwined and superimposed is a feasible method to predict the residual deformation of foam. This new modeling approach can more accurately describe the mechanical response of foams under inhomogeneous compression conditions than the traditional theoretical models. In this study, the mechanical behavior of foams under a single compressive impact load was mainly investigated. In the future, this hierarchical modeling method can also be applied to study the mechanical response of foams under continuous compressive impact loading.

## Figures and Tables

**Table 1:** Summary of foam model parameters for finite element analysis.

Foam Type	Loading Situation	*μ*_1_/MPa	*α*_1_	*μ*_2_/MPa	*α*_2_	*r*	*m*/MPa	*β*	*E*/MPa	λ
EPE29	Static	0.04	0.69	--	--	1.5	0.004	0.9	0.14	0.34
	Dynamic	0.046	0.46	--	--	1.5	0.004	0.9	0.16	0.34
EPE36	Static	0.65	29.90	0.015	0.23	1.2	0.003	0.8	1.01	0.4
	Dynamic	1.44	32.99	0.003	0.03	1.2	0.003	0.4	2.46	0.4
EPE58	Static	1.83	40.0	0.014	0.01	1.2	0.002	0.5	3.49	0.61
	Dynamic	2.76	27.89	−0.09	4	1.2	0.002	0.5	4.08	0.61

## Data Availability

No new data were created.
